# Aldose reductase inhibitory activity and antioxidant capacity of pomegranate extracts

**DOI:** 10.2478/v10102-012-0003-8

**Published:** 2012-03

**Authors:** Çimen Karasu, Ahmet Cumaoğlu, Ali Rifat Gürpinar, Murat Kartal, Lucia Kovacikova, Ivana Milackova, Milan Stefek

**Affiliations:** 1 Cellular Stress Response & Signal Transduction Research Laboratory, Gazi University, Faculty of Medicine, Ankara, Turkey; 2 Department of Pharmacognosy, Ankara University, Faculy of Pharmacy, Ankara, Turkey; 3 Institute of Experimental Pharmacology & Toxicology, Slovak Academy of Sciences, Bratislava, Slovak Republic

**Keywords:** Pomegranate, aldose reductase inhibition, antioxidant, diabetic complications

## Abstract

The pomegranate, *Punica granatum* L., has been the subject of current interest as a medicinal agent with wide-ranging therapeutic indications. In the present study, pomegranate ethanolic seed and hull extracts were tested, in comparison with a commercial sample, for the inhibition of aldose reductase, an enzyme involved in the etiology of diabetic complications. In vitro inhibition of rat lens aldose reductase was determined by a conventional method. Pomegranate ethanolic hull extract and commercial pomegranate hull extract exhibited similar aldose reductase inhibitory activity characterized by IC_50_ values ranging from 3 to 33.3 μg/ml. They were more effective than pomegranate ethanolic seed extract with IC_50_ ranging from 33.3 to 333 μg/ml. Antioxidant action of the novel compounds was documented in a DPPH test and in a liposomal membrane model, oxidatively stressed by peroxyl radicals. All the plant extracts showed considerable antioxidant potential in the DPPH assay. Pomegranate ethanolic hull extract and commercial pomegranate hull extract executed similar protective effects on peroxidatively damaged liposomal membranes characterized by 10<IC_50_<100 μg/ml. Pomegranate ethanolic seed extract showed significantly lower antioxidant activity compared to both hull extracts studied. Pomegranate extracts are thus presented as bifunctional agents combining aldose reductase inhibitory action with antioxidant activity and with potential therapeutic use in prevention of diabetic complications.

**LIST OF ABBREVIATIONS**
AAPH: 2,2'-azobis(2-amidinopropane) hydrochloride
AO: antioxidant
ALR2: aldose reductase
ARI: aldose reductase inhibitor
BHT: 2,6-di-tert-butyl-p-cresol
t-BuOOH: tert-butyl hydroperoxide
CPHE: Commercial pomegranate hull extract
DOPC: L-α-phosphatidylcholine dioleoyl (C18:1, [cis]-9)
DPPH: 1,1'-diphenyl-2-picrylhydrazyl
I(%): percentage of inhibition
PEHE: Pomegranate ethanolic hull extract
PESE: Pomegranate ethanolic seed extract

## Introduction

Over the past few decades, scientific research has provided credible evidence for multiple uses of pomegranate in traditional ethnomedicine. Significant progress has been made in establishing the pharmacological mechanisms responsible for beneficial effects of pomegranate (Grover *et al.*, 2002; Lansky & Newman, 2007; Katz *et al.*, 2007; Jurenka, 2008; Bell & Hawthorne, 2008; Wang *et al.*, 2010). The therapeutically most beneficial pomegranate constituents appear to be ellagic acid, ellagitannins, punicic acid, flavonoids, anthocyanidins, anthocyanins, and estrogenic flavonols and flavones (Jurenka, 2008; El Kar *et al.*, 2011). Ellagic acid was reported to exhibit powerful anticarcinogenic and antioxidant properties, propelling it to the forefront of pomegranate research (Bell & Hawthorne, 2008). At the same time, the synergistic action of the pomegranate constituents should be taken into consideration since it may overcome the effects of single constituents (Lansky, 2006).

The traditional folk medicines of India describe antidiabetic effects of pomegranate (Jafri, 2000; Das *et al.*, 2001; Grover *et al.*, 2002; Li *et al.*, 2005; Katz *et al.*, 2007; Bagri *et al.*, 2009; Jurenka, 2008). The mechanisms of hypoglycemic activity are largely unknown, though recent research suggests that pomegranate may prevent diabetic sequelae via peroxisome proliferator-activated receptor-gamma binding and nitric oxide production (Huang *et al.*, 2005; Katz *et al.*, 2007; Li *et al.*, 2008; Hontecillas *et al.*, 2009).

Pomegranate extracts have also the potential to attenuate diabetic complications via their ability to inhibit posttranslational modifications of proteins based on their antioxidant (Gil *et al.*, 2000; Chidambara *et al.*, 2002; Singh *et al.*, 2002; Cerdá *et al.*, 2004; Seeram *et al.*, 2005; Jurenka, 2008; Zahin *et al.*, 2010; Dikmen *et al.*, 2011; Joseph *et al.*, 2011; Elfalleh *et al.*, 2011) and antiglycation (Rout & Banerjee, 2007) activities. In addition, considering ellagic acid, quercetin and other flavonol content, pomegranate may affect the polyol pathway – another key mechanism involved in the etiology of diabetic complications. Aldose reductase (ALR2), the first enzyme of the polyol pathway, catalyzes the reduction of glucose to sorbitol. At normoglycemic conditions, less than 3% of glucose turns to sorbitol but in hyperglycemia more than 30% of glucose undergoes the polyol pathway, which results in accumulation of sorbitol in tissues where glucose uptake is insulin independent (Kador, 1998; Yabe-Nishimura, 1998; Kyselova, 2004; Alexiou *et al.*, 2009). This excessive sorbitol accumulation may result in disruption of cellular osmotic homeostasis (Kador *et al.*, 2000; Del Corso *et al.*, 2008). In addition, the increased flux of glucose through the polyol pathway and consequent depletion of NADPH may inhibit the activity of other NADPH-requiring enzymes, including those of the glutathione redox cycle. In turn, the decreased levels of reduced glutathione increase the susceptibility of cells to damage by oxidative stress (Hamada *et al.*, 1996; Obrosova, 2005). The above-mentioned processes related to hyperglycemia are considered to be key steps in the development of diabetic complications, including macro- and microvasculopathies, neuropathy, cataract, retinopathy and nephropathy (Kador, 1998; Yabe-Nishimura, 1998; Alexiou *et al.*, 2009; Kador *et al.*, 2000; Del Corso *et al.*, 2008; Oates 2008).

Pharmacological use of antioxidants (AOs) and aldose reductase inhibitors (ARIs) has been recognized as an important strategy in the prevention and attenuation of long-term diabetic complications (Coudert *et al.*, 1994; Constantino *et al.*, 1999; Scott & King, 2004; Stefek *et al.*, 2008; Alexiou *et al.*, 2009; Juranek *et al.*, 2009). Involvement of the polyol pathway and oxidative stress in the etiology of diabetic complications requires inhibition of both processes. Therefore, bifunctional compounds with joint antioxidant/aldose reductase inhibitory (AO/ARI) activities would be dually beneficial.

In this study, we compared AO/ARI activities of two ethanolic pomegranate extracts with those of a commercially available pomegranate extract standardized to 40% ellagic acid.

## Material and methods

### Plant materials and extracts

The dried seeds and hull of *Punica granatum* (20 g) were extracted with 200 ml of ethanol (Sigma, Aldrich) by occasional stirring at 40°C. The ethanol phases were filtered and dried under vacuum by using rotary-evaporator to give the crude extract. The commercial Punica granatum hull extract, standardized to 40% ellagic acid content, was obtained from Refine Biology (China) via LongAge Health Company (Turkey).

### Other chemicals

2,2‘-Azobis(2-amidinopropane)hydrochloride (AAPH) was obtained from Fluka Chemie GmbH (Buchs, Switzerland). Egg yolk L-α-phosphatidylcholine dioleoyl (C18:1, [cis]-9) (DOPC) (99% grade), 2,6-di-t-butyl-p-cresol (BHT), 1,1’-diphenyl-2-picrylhydrazyl (DPPH) NADPH and D,L-glyceraldehyde were obtained from Sigma Chemical Co. (St. Louis, MO, USA). Other chemicals were purchased from local commercial sources and were of analytical grade quality. All solvents used for lipid peroxidation studies were deareated under nitrogen.

### Determination of ellagic acid content

Ellagic acid content of extracts was determined with Liquid Chromatography (Agilent Technologies 1200 Series High Pressure Liquid Chromatography, including a binary pump, vacuum degasser, autosampler, diode array detector). Chromatographic separations were performed on Eclipse XDB-C18 column (15 cm×4.6 mm, 5 μm). A mobile phase consisting of two eluents, (A) acetonitrile and (B) 40 mM formic acid, was used for separation with a gradient elution. The flow rate was 1.0 ml/min and compounds were detected at 254 nm. The injection volume was 10 μl. All the calculations concerning the quantitative analysis were performed with external standardization by measurement of peak areas.

### DPPH free radical scavenging assay

To investigate the antiradical activity of the pomegranate extracts, the ethanolic solution of DPPH (50 μM) was incubated in the presence of an extract tested (300 μg/ml) at laboratory temperature. The absorbance decrease, recorded at 518 nm, during the first 15-s interval was taken as a marker of antiradical activity.

### Preparation of ALR2 enzyme

Rat lens ALR2 was partially purified using a procedure adapted from Hayman and Kinoshita (1965) as follows: lenses were quickly removed from rats following euthanasia and homogenized in a glass homogenizer with a teflon pestle in 5 vol. of ice-cold distilled water. The homogenate was centrifuged at 10,000×g at 0–4°C for 20 min. The supernatant was precipitated with saturated ammonium sulfate (Sigma Aldrich) at 40, 50% and then at 75% salt saturation. The supernatant was retained after the first two precipitations. The pellet from the last step, possessing ALR2 activity, was dispersed in 75% ammonium sulfate and stored in smaller aliquots in liquid nitrogen container.

### ALR2 enzyme assay

ALR2 activities were assayed spectrophotometrically by determining NADPH consumption at 340 nm and were expressed as decrease of the optical density. To determine ALR2 activity (Da Settimo *et al.*, 2005), the reaction mixture contained 4.67 mM D,L-glyceraldehyde (Sigma Aldrich) as a substrate, 0.11 mM NADPH (Sigma Aldrich), 0.067 M phosphate buffer, pH 6.2 and 0.05 ml of the enzyme preparation in a total volume of 1.5 ml. The reference blank contained all the above reagents except the substrate D,L-glyceraldehyde to correct for the oxidation of NADPH not associated with reduction of the substrate. The enzyme reaction was initiated by addition of D,L-glyceraldehyde and was monitored for 4 min after an initial period of 1 min at 30°C. Enzyme activity was adjusted by diluting the enzyme preparation with buffer so that 0.05 ml of the preparation gave an average reaction rate for the control sample of 0.02±0.005 absorbance units/min. The effect of extracts on the enzyme activity was determined by including each sample at required concentration to the reaction mixture. The extract was included in the reference blank in the same concentration.

#### Liposome preparation and incubation.

DOPC (15.7 mg) was placed in a round-bottom flask and dissolved in chloroform (5 ml). The solvent was subsequently removed under nitrogen, and the resulting thin film on the walls was dispersed in phosphate buffer (20 ml, 20 mM, pH 7.4) by vigorous stirring for 2 min followed by sonication for the same period of time. A suspension of unilamellar liposomes (1 mM DOPC) was thus obtained. The liposomes (final concentration 0.8 mM DOPC) were incubated in the presence of different concentrations of the extracts tested with the water-soluble initiator AAPH (final concentration 10 mM) at 50°C for 80 min. Aliquots (1 ml) of the incubation mixtures were extracted by 2 ml portions of ice-cold mixture CHCl_3_/MeOH (2:1,v/v) containing BHT (0.05%). Lipid hydroperoxide content was determined by thiocyanate method according to Mihaljevic *et al.*, (1996) by sequentially adding CHCl_3_/MeOH (2:1, v/v) mixture (1.4 ml) and the thiocyanate reagent (0.1 ml) to 1-ml aliquots of the liposome extracts. The reagent was prepared by mixing equivalent volumes of methanolic solution of KSCN (3%) and ferrous-ammonium sulfate solution (45 mM in 0.2 mM HCl). After leaving the mixture at ambient temperature for at least 5min, the absorbance at 500 nm was recorded by Hewlett–Packard Diode Array Spectrophotometer 8452A.

## Results

Liquid chromatography analysis of the pomegranate extracts showed the content of ellagic acid as follows: pomegranate seed extract 21.48±2.24% and pomegranate hull extract 20.34±3.90%.

**Figure 1:**
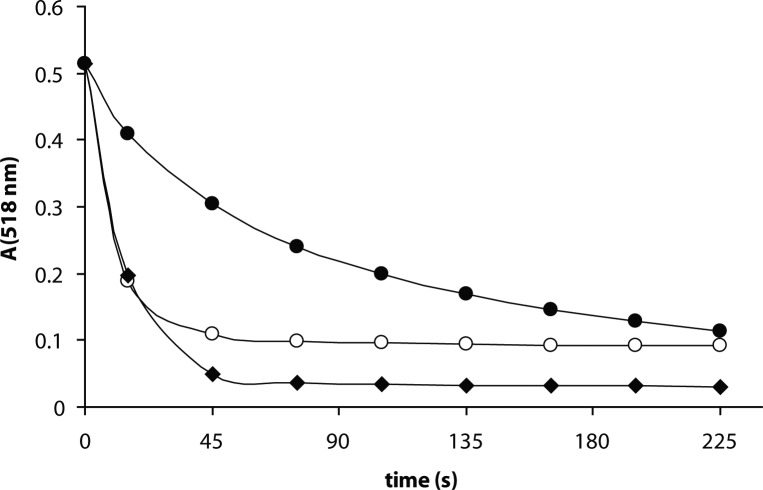
Free radical scavenging activity of pomegranate extracts in a DPPH assay. Time dependence. The ethanolic solution of DPPH radical (50 μM) was incubated in the presence of the extracts (300 μg/ml). (○)-Commercial pomegranate hull extract (CPHE); (●)-Pomegranate ethanolic seed extract (PESE); (♦)-Pomegranate ethanolic hull extract (PEHE). Results of three typical experiments.

In order to determine the radical-scavenging potential of the extracts tested, the reactivity toward the stable free radical DPPH was measured by continual absorbance decrease of ethanol solution of DPPH (50 μM) containing the samples tested (300 μg/ml) at 518 nm (Figure 1). Initial rates of absorbance decrease were determined during the first 15s interval and compared with the effect of the reference antioxidant trolox (12.5 μg/ml). As shown in Table 1, the antiradical activity of the extracts increased in the order: pomegranate ethanolic seed extract (PESE)<pomegranate ethanolic hull extract (PEHE)<commercial pomegranate hull extract (CPHE).

**Table 1:** Antiradical activities of pomegranate extracts in aDPPH test in comparison with the standard trolox.

Extract	Absorbance decrease (-ΔA/15s)
CPHE (300 μg/ml)	0.326±0.015
PESE (300 μg/ml)	0.105±0.054
PEHE (300 μg/ml)	0.317±0.016
Trolox (12.5 μg/ml)	0.388±0.019

CPHE, Commercial pomegranate hull extract; PEHE, Pomegranate ethanolic hull extract; PESE, Pomegranate ethanolic seed extract. Experimental results are mean values±SD from at least three experiments.

The extracts were evaluated for their ability to inhibit the *in vitro* reduction of D,L-glyceraldehyde by partially purified ALR2 from the rat lens. As shown in Table 2, commercial pomegranate extract (CPHE) and pomegranate ethanolic hull extract (PEHE) showed similar inhibitory activities toward ALR2 with estimated values of 50% inhibition as follows: 3 μg/ml<IC_50_<33.3 μg/ml. These extracts were more effective than pomegranate ethanolic seed extract (PESE) with IC_50_ ranging from 33.3 to 333 μg/ml.

**Table 2:** Inhibitory activity of pomegranate extracts toward ALR2.

	I(%) at concentration		
Extract	3 μg/ml	33.3 μg/ml	333 μg/ml
CPHE	36.11±5.4	95.89±9.1	−
PESE	−	36.58±6.0	96.14±8.9
PEHE	16.18±2.4	72.64 ±7.3	−

Percentage inhibition values of aldose reductase I(%) were determined at given concentrations of the extracts. CPHE, Commercial pomegranate hull extract; PEHE, Pomegranate ethanolic hull extract; PESE, Pomegranate ethanolic seed extract. Experimental results are mean values±SD from at least three experiments.

In our further experiments, the overall antioxidant action of the extracts was determined in the model of unilamellar dioleoyl L-α-phosphatidylcholine (DOPC) liposomes. Peroxidation of liposomes was induced by a water-soluble radical generator, 2,2′-azobis(2-amidinopropane)hydrochloride (AAPH), which simulates an attack by peroxyl radicals from the aqueous region. In acomplete reaction system, DOPC liposomes/AAPH/buffer, lipid peroxidation proceeded at aconstant rate and approximately alinear time-dependent increase of lipid hydroperoxides was observed during the first 80-min interval. No accumulation of hydroperoxides was observed in the absence of AAPH or liposomes. As shown in Table 3, commercial pomegranate hull extract (CPHE) and pomegranate ethanolic hull extract (PEHE) executed similar protective effects on peroxidatively damaged liposomal membranes characterized by 10<IC_50_ <100 μg/ml. On the other hand, the antioxidant action of pomegranate ethanolic seed extract (PESE) was characterized by IC_50_ value ranging from 100 to 500 μg/ml.

**Table 3:** Inhibitory activity of pomegranate extracts against AAPH-induced peroxidation of DOPC liposomes.

	I(%) at concentration		
Extract	10 μg/ml	100 μg/ml	500 μg/ml
CPHE	32.7±2.6	96.8±7.7	−
PESE	−	8.7±0.9	91.3±8.2
PEHE	4.1±0.6	85.4±8.8	−

Percentage inhibition values I(%) were determined for the inhibition of AAPH-induced peroxidation of DOPC liposomes at the time interval of 80min. DOPC liposomes (0.8 mM) were incubated in the presence of AAPH (10 mM) in phosphate buffer (20 mM; pH 7.4) at 50°C. CPHE, Commercial pomegranate hull extract; PEHE, Pomegranate ethanolic hull extract; PESE, Pomegranate ethanolic seed extract. Experimental results are mean values±SD from at least three experiments.

## Discussion

The potential therapeutic uses of pomegranate appear to be wide-ranging. Extracts of all parts of the fruit were reported to have therapeutic properties including anti-inflammatory, anticancer, anti-atherogenic, antidiabetic, antibacterial, anti-glycation, etc. activities (Jurenka, 2008; Lansky & Newman, 2007; Katz & Lansky, 2007; Naz *et al.*, 2007; Rout & Banerjee, 2007; Wang *et al.*, 2010).

Recently great progress has been made in disclosing the molecular mechanisms responsible for the multiple medicinal actions of pomegranate. Antioxidant action of pomegranate juice and extracts, attributed to its high content of polyphenols like ellagitannins and ellagic acid, has been well documented (Gil *et al.*, 2000; Chidambara *et al.*, 2002; Singh *et al.*, 2002; Cerdá *et al.*, 2004; Kulkarni *et al.*, 2004; Seeram *et al.*, 2005; Han *et al.*, 2006; Jurenka, 2008; Zahin *et al.*, 2010; Dikmen *et al.*, 2011; Joseph *et al.*, 2011; Elfalleh *et al.*, 2011). Although the purified polyphenols, including ellagic acid, showed significant antioxidant effects alone, the superior activity of the whole pomegranate juice suggests synergistic and/or additive effects from the other phytochemicals present (Seeram *et al.*, 2005).

On the other hand, information of aldose reductase (ALR2)-inhibitory activity of pomegranate is scarce. To our knowledge, there is only one study reporting on this phenomenon. Recently Sun *et al.* (2008) described the inhibitory effect of polyphenol extracts from pomegranate peel on aldose reductase activity. More data is available on ALR2-inhibitory action of ellagic acid, one of the key constituents of pomegranate extracts (Ueda *et al.*, 2004). Quercetin and some other flavonoids represent further pomegranate constituents endowed with ALR2-inhibitory action (Stefek, 2011; Stefek & Karasu, 2011).

The aim of this study was to compare antioxidant and ALR2-inhibitory action of two ethanolic pomegranate extracts with a commercially available pomegranate extract standardized to 40% content of ellagic acid.

In agreement with literary data, significant antioxidant action was recorded for the extracts studied, based on DPPH and liposome tests. DPPH, as a weak hydrogen atom abstractor, is considered a good kinetic model for peroxyl ROO^•^ radicals (Blois, 1958). An absorbance decrease of DPPH at 518 nm, shown in Figure 1, was used as a measure of the antiradical activity of the samples tested. As shown in Table 1, the antiradical activity of the extracts, expressed as the initial velocity of the absorbance decrease calculated for the first 15 s, increased in the order PESE<PEHE<CPHE. For comparison, antiradical activity of standard trolox present at a24-times lower mass content is presented. Based on I(%) values at the given concentrations, the order of antioxidant activities of the extracts determined in the liposome test was PESE<PEHE<CPHE. In agreement with the DPPH test, PESE showed the lowest antioxidant activity. Analogically, Singh *et al.* (2002) reported significantly lower antioxidant activity of pomegranate methanolic seed extracts compared to peel extracts.

In a homogeneous system of DPPH in ethanol, antioxidant activity stems from an intrinsic chemical reactivity towards radicals. In heterogeneous systems comprising membranes, however, the relative reactivity may be different since it is determined also by additional factors, such as mutual location of the antioxidant and radicals, ruled predominantly by their partition ratios between water and lipid compartments. Interestingly, the order of intrinsic antiradical activities from the DPPH test was identical also for the overall antioxidant activities of the extracts observed in liposomes.

We found IC_50_ values for inhibition of ALR2 by CPHE and PEHE to be less than 33.3 μg/ml. These results correspond well with the data reported by Sun *et al.* (2008). In their study of ALR2-inhibitory action of the pomegranate peel using aldose reductase from bovine lens, acetone extract demonstrated the strongest inhibition followed by methanol and water extracts, their respective 50% inhibition concentrations being 34.77, 44.18 and 62.07 μg/ml. In our study, a significantly lower inhibition was recorded for PESE with IC_50_ between 33.3 and 333 μg/ml. Considering the fact that the content of ellagic acid, the component of high ALR2-inhibitory potential, was found to be similar in PEHE and PESE, the higher inhibitory activity of PEHE may be accounted for by the presence of additional constituent(s) of high inhibitory potential. Potential candidates to be considered are quercetin, rutin, and other flavonols, with high ALR2-inhibitory activity (Stefek, 2011), whose presence in pomegranate peel, in contrast to seeds, is well documented (Artik, 1998; Naz *et al.*, 2007; Jurenka, 2008).

## Conclusion

Aldose reductase inhibition and elimination of consequences of oxidative stress has been recognized as important strategy in the prevention and attenuation of long-term diabetic complications. In view of the multiple biochemical activities of pomegranate extracts, we believe that they deserve further investigations as potential multitarget-oriented remedies for diabetes mellitus and its pathological consequences.
